# Racial and Ethnic Differences in Hospitalizations of Incarcerated Patients

**DOI:** 10.1007/s40615-025-02562-y

**Published:** 2025-07-25

**Authors:** Farah Acher Kaiksow, Marguerite E. Burns, Amir Forati

**Affiliations:** 1 Department of Medicine, Division of Hospital Medicine, University of Wisconsin School of Medicine and Public Health, Madison, WI 53792 USA; 2 Department of Population Health Sciences, University of Wisconsin School of Medicine and Public Health, Madison, WI 53792 USA; 3 Department of Medicine, University of Wisconsin School of Medicine and Public Health, Madison, WI 53792 USA

**Keywords:** Incarceration, Hospitalization, Disproportionality

## Abstract

There are significant racial and ethnic disparities in the incarcerated population in the USA, with individuals from racial and ethnic minoritized groups more likely to be incarcerated. There are also disparities in the health and health care received by different racial and ethnic groups in the USA. The information on the health care received by incarcerated individuals is lacking, including particularly limited data on hospitalization rates. This paper aims to identify potential racial and ethnic differences in hospital care provided to patients who are incarcerated. Differences in care during incarceration may worsen pre-existing disparities between racial and ethnic groups.

## Background

There are significant racial and ethnic disparities in who is incarcerated in the USA.^1^ In 2022, Black/African American people made up 14% of the US population but 42% of those incarcerated. American Indian/Alaska Native people represented a much smaller part of the population at 1% but were also disproportionately incarcerated at 3% of the incarcerated population [1]. While there is an abundance of evidence regarding differences in the care provided to minoritized groups in the USA [2], data on the provision of health care and associated differences for incarcerated patients are limited, including whether differences in hospitalization patterns might be a source of health disparities.

## Objective

To identify potential differences in access to hospital care by comparing the racial and ethnic composition of incarcerated patients who were hospitalized with the overall incarcerated population of the state.

## Methods

We collected race and ethnicity data for patients who were male, at least 18 years old, and admitted to a tertiary care academic hospital for any length of time while in the custody of the state’s Department of Corrections (DOC) between January 1, 2010 and December 31, 2019. Because there are meaningful differences in the characteristics of males and females who experience incarceration [3], and because males make up approximately 90% of all incarcerated people in the USA, we chose to limit our study to male patients only. Patients were admitted through the emergency department (ED) or a telephone triage process. We collected the Charlson Comorbidity Index (CCI) as a surrogate measure for severity of illness [4]. The study hospital is the state’s largest provider of inpatient care for DOC patients. Using average end-of-year population data from the state DOC for 2010–2019, we estimated the racial and ethnic composition of the overall incarcerated population. To evaluate racial differences within the hospitalized incarcerated population, we calculated a disproportionality index (DI), representing the ratio of the proportion of a specific racial or ethnic demographic in the hospitalized population to that in the reference incarcerated population. The analysis was conducted at the admission level. A chi-square goodness-of-fit test compared the observed admission counts with their expected values under the DOC census proportions. Significance was set at *α* = 0.05. Finally, to test whether illness severity might explain utilization differences, we compared mean CCI scores across racial groups using the Kruskal–Wallis test.

## Findings

Our dataset included 4525 admissions. During the study years, the DOC population in the state was 52.8% White, 42.0% Black/African American, and 4.1% American Indian/Alaska Native; Latine people made up 10% of the incarcerated population. In comparison, 63.6% of hospitalizations from the DOC were for White patients, 31.4% were for Black/African American patients, and 2.7% were for American Indian/Alaska Native patients. Latine patients were involved in 6.1% of admissions. Asian American people were excluded from the analysis as they did not make up a substantial proportion of the state’s DOC population. Mean CCI scores did not differ significantly among White, Black/African American, and American Indian/Alaska Native groups (Kruskal–Wallis *p* = 0.76). The mean CCI for Latinx patients (4.27) exceeded those of other groups, suggesting greater illness severity in that subgroup.

The chi-square test showed a significant divergence between observed and expected racial distributions of hospitalizations (*χ*^2^ = 242.9, 2 df, *p* < 0.001). White patients were admitted 20% more often than expected, whereas Black/African American patients were hospitalized 25% less often, and American Indian/Alaska Native patients were hospitalized 34% less often than expected. These results are shown in Table 1.

**Table 1:** Charlson Comorbidity Index (CCI), proportionality data, and disproportionality indices

Race and ethnicity	Average CCI^1^	Percent of total incarcerated population	Percent of incarcerated patient admissions	DI (95% CI)
American Indian/Alaska Native	3.84	4.1%	2.7%	0.66 (0.54–0.77)
Black/African American	3.85	42.0%	31.4%	0.75 (0.72–0.78)
Latine	4.27	10.0%	6.1%	0.61 (0.54–0.69)
White	3.84	52.8%	63.6%	1.20 (1.18–1.23)

^1^Kruskal–Wallis *p* = 0.76

## Discussion

In this 10-year data set of admissions of patients from the state DOC to an academic hospital, we found racial and ethnic differences in frequency of admission. Incarcerated patients from minoritized groups, including American Indian/Alaska Native, Black/African American, and Latine, were underrepresented in hospital admissions based on their proportions within the total DOC population. Our findings may be a marker of bias within the carceral system; health care providers from the DOC make decisions on who should be transferred to the hospital. It may also represent bias within the hospital system as hospital system providers perform triage for incarcerated patients through the telephone triage system and ED visits.

This disproportionality cannot be explained by differing levels of illness severity as the CCI for American Indian/Alaska Native, Black/African American, and White populations were similar, and the CCI for Latine patients was higher than the other groups. The study state has some of the starkest racial and ethnic disparities in medical care and health outcomes, with American Indian/Alaska Native and Black/African American people experiencing the most significant inequities [2, 5]. These minoritized groups in state prisons could thus reasonably be expected to have poorer health than other groups, and therefore require more frequent hospital care. Additionally, the higher CCI for the Latine population raises the question of delayed care for this marginalized population; are incarcerated Latine individuals hospitalized only when they are sicker than others?

Our study has limitations. The CCI is an imperfect measure for comorbidities as it excludes many conditions, particularly mental health, substance use, and certain infectious diseases, which are all known to have high prevalence in justice-involved individuals [6]. The number of individuals included in the American Indian/Alaska Native group was small (*n* = 122); however, the DI confidence interval excluded one, indicating statistically significant under-representation even after accounting for sampling variability. The width of that interval (± 0.11) reflects the limited sample and suggests caution when applying our findings to other populations, yet it still supports a meaningful signal that aligns with the broader pattern observed for Black/African American patients*.* Our analysis did not include age, and future work should incorporate age data to determine whether racial differences persist once adjusted for age; however, as mean CCI scores were similar across racial groups, age differences alone are unlikely to account for the hospitalization gap. Remaining unmeasured factors—such as sentence length and facility‐level policies—could not be addressed using our dataset and should be pursued in future research. Finally, our study excluded female incarcerated patients and so should not be used to extrapolate to that unique population.

In the USA, people from American Indian/Alaska Native, Black/African American, and Latine populations are more likely to be incarcerated than other groups [7, 8]. People from these minoritized groups are also more likely to receive poor medical care and have poor health [2]. Our findings suggest that differential rates of hospitalizations may be one mechanism by which preexisting racial and ethnic health disparities are exacerbated during incarceration.

As the incarcerated population continues to age, there is an urgent need to develop interventions to both improve the overall quality of care received by these patients and to address disparities in their care delivery. Solutions to this major public health problem will necessarily be multifaceted. Increased data availability and access to carceral care systems are necessary, though not sufficient, parts of the solution. A potential policy adjustment may be to shift the responsibility for the health care for people in state prisons from state Departments of Corrections to state Departments of Health. Ultimately, policymakers and partners from both the health and the carceral space will need to work together to address the challenges of providing high-quality and equitable care to incarcerated individuals in the USA.

## Data Availability

Fully deidentified data will be made available within a year of the project’s completion through openICPSR.

## Footnotes

1 The authors acknowledge that race and ethnicity are social constructs, not biological variables. We use them here as a marker of potential discrimination, rather than a biological determinant of health.
